# Dietary patterns and chronic prostatitis: a symptom severity prediction model based on nutritional clustering and machine learning

**DOI:** 10.3389/fnut.2025.1660430

**Published:** 2026-01-19

**Authors:** Zhen Wang, Wei Wu, Bo Wen, Zhongle Xu, Junhua Xi, Yanbin Zhang

**Affiliations:** Department of Urology, Hefei Second People’s Hospital, Hefei, Anhui, China

**Keywords:** chronic prostatitis, dietary pattern, food frequency questionnaire, machine learning, principal component analysis, LASSO regression, XGBoost, SHAP interpretation

## Abstract

**Background:**

Chronic prostatitis/chronic pelvic pain syndrome (CP/CPPS) has a multifactorial etiology where diet is considered an important factor. This study aimed to develop a predictive model for CP/CPPS symptom severity by analyzing food frequency questionnaire (FFQ) data with machine learning techniques, providing a basis for personalized nutritional interventions.

**Methods:**

This study included 313 patients with CP/CPPS. We used principal component analysis (PCA) to extract dietary patterns from FFQ data and applied LASSO regression to select key predictors of symptom severity. Subsequently, six machine learning models (logistic regression, random forest, XGBoost, support vector machine, K-nearest neighbors, and multilayer perceptron) were trained and compared. Model performance was evaluated using ROC curves, decision curve analysis (DCA), and calibration plots. SHapley Additive exPlanations (SHAP) were used to interpret the optimal model.

**Results:**

PCA identified two major dietary patterns: a “Red Meat and Processed Food” dietary pattern (PC1) and a “Dairy-rich” pattern (PC2). LASSO regression selected key predictors, among which the “Red Meat and Processed Food” dietary pattern demonstrated the strongest positive association with CP/CPPS symptom severity. Among the models, while support vector machine (SVM) and logistic regression showed high AUC values, the XGBoost model demonstrated the best overall performance across a balance of metrics including accuracy, precision, recall, and F1-score, and was selected as the final model (AUC = 0.883). SHAP analysis identified the Red Meat and Processed Food dietary pattern as the most important feature associated with symptom severity.

**Conclusion:**

This study successfully developed a machine learning model based on dietary patterns that effectively predicts CP/CPPS symptom severity. The model underscores the significant association between nutrition and disease management and, with its strong predictive performance and interpretability, offers a novel tool for precision nutrition in CP/CPPS.

## 1 Introduction

Chronic prostatitis/chronic pelvic pain syndrome (CP/CPPS) is a prevalent condition marked by pelvic pain, lower urinary tract symptoms, and reduced quality of life, placing a substantial burden on men’s health (1). Although the pathogenesis of CP/CPPS remains unclear, it is widely thought to involve multiple pathophysiological mechanisms, such as inflammation, neuroimmune dysregulation, oxidative stress, and psychosocial influences (2, 3). Growing evidence highlights that lifestyle factors, particularly dietary habits, play a significant role in the development and progression of CP/CPPS (4, 5).

The influence of dietary patterns on prostate health has become a prominent research focus. For example, the “Western” dietary pattern—characterized by high consumption of red meat, processed foods, refined grains, and sugary drinks—has been linked to an increased risk of chronic diseases, including prostate cancer, primarily due to its pro-inflammatory and pro-oxidative effects (6, 7). In contrast, healthy dietary patterns—rich in vegetables, fruits, whole grains, fish, and legumes—may offer protective effects on prostate health through anti-inflammatory and antioxidant pathways (8, 9). However, traditional nutritional epidemiology faces notable limitations in evaluating the relationship between diet and disease. Focusing solely on individual nutrients or food groups may neglect complex synergistic and antagonistic interactions among dietary components, thereby limiting the ability to assess the impact of overall dietary patterns. Moreover, the dependence on precise quantification of nutrient intake in grams may introduce recall bias and increase the complexity of data collection (10).

In recent years, the emergence of big data and artificial intelligence has presented new opportunities for advancing nutritional epidemiology. Machine learning algorithms can detect hidden patterns and nonlinear relationships within high-dimensional dietary data, thereby enhancing the accuracy of disease risk and symptom severity prediction (11, 12). Specifically, integrating food frequency questionnaire (FFQ) data with machine learning techniques enables the effective capture of long-term dietary behaviors and the development of clinically applicable predictive models (13).

This study aims to construct a predictive model of CP/CPPS symptom severity using dietary data derived from FFQs. Principal component analysis (PCA) was applied to extract dietary patterns, followed by feature selection using the Least Absolute Shrinkage and Selection Operator (LASSO) regression. Multiple machine learning algorithms were subsequently compared in terms of their ability to predict CP/CPPS symptom severity. Model performance was thoroughly evaluated using receiver operating characteristic (ROC) curves, decision curve analysis (DCA), calibration curves, and SHapley Additive exPlanations (SHAP), with the ultimate goal of developing a clinically applicable tool to support personalized dietary interventions in patients with CP/CPPS.

## 2 Materials and methods

### 2.1 Study population

This retrospective study enrolled patients with CP/CPPS who attended the urology outpatient clinic of our hospital between January 2022 and December 2023. The inclusion criteria were as follows: age between 18 and 60 years; a diagnosis of CP/CPPS confirmed using the National Institutes of Health Chronic Prostatitis Symptom Index (NIH-CPSI); symptom duration exceeding 3 months; and voluntary participation with written informed consent. Exclusion criteria included: acute bacterial prostatitis; other prostatic diseases such as prostate cancer or benign prostatic hyperplasia; urinary tract infections; other identifiable causes of pelvic pain; and severe systemic or psychiatric conditions that might interfere with questionnaire completion. The study protocol was approved by the Hefei Second People’s Hospital Institutional Ethics Committee (Approval No.: 2025-KY-052). Written informed consent was obtained from all participants prior to enrollment.

### 2.2 Data collection

Patient data were collected using standardized questionnaires and electronic medical records, including the following components:

- Demographic data: Age (years), height (cm), weight (kg), and body mass index (BMI, kg/m^2^), which was calculated from height and weight. Lifestyle variables, including smoking status (yes/no), alcohol consumption (yes/no), and physical activity level (low, moderate, or high), were also recorded.
- Symptom assessment: CP/CPPS symptoms were assessed using the National Institutes of Health Chronic Prostatitis Symptom Index (NIH-CPSI), which comprises nine items spanning three domains: pain (0–21 points), urinary symptoms (0–10 points), and quality of life impact (0–15 points). The total score ranges from 0 to 46, with higher scores reflecting greater symptom severity. In this study, the total NIH-CPSI score was used as the primary outcome. For predictive modeling, the symptom severity was dichotomized into “mild” (score 0-14) and “moderate-to-severe” (score ≥ 15) categories. This cutoff is widely adopted in clinical studies of CP/CPPS to distinguish different levels of symptom burden and has been validated for its clinical relevance (doi: 10.1016/j.juro.2008.06.06, DOI: 10.1016/j.eururo.2015.08.06).
- Dietary assessment: A semi-quantitative food frequency questionnaire (FFQ), covering approximately 100 commonly consumed food items, was used to evaluate dietary intake over the previous year. Patients reported the frequency of consumption for each item using nine categorical options ranging from “never or rarely” to “more than four times per day.” These categorical frequencies were converted into numerical values (scored 0–3) for dietary pattern extraction. Detailed variable definitions and coding schemes are provided in Supplementary Table S1.

### 2.3 Dietary pattern extraction

Principal component analysis (PCA), a multivariate statistical method, was applied to extract meaningful dietary patterns from high-dimensional FFQ data. PCA transforms the original variables into a set of orthogonal principal components that retain the maximum possible variance of the original data (14). In this study, the FFQ data were standardized prior to PCA, and two principal components—Dietary_Pattern_PC1 and Dietary_Pattern_PC2—were extracted. The components were interpreted and labeled according to the factor loadings of individual food groups. Positive factor loadings indicate a direct association with the component, whereas negative loadings reflect an inverse relationship.

Dietary_Pattern_PC1 was characterized by high intakes of red meat, fried foods, sugary beverages, and processed meats, along with low intakes of vegetables, fruits, and whole grains. This pattern, while reflecting a similar nutritional structure (high in unhealthy fats and sugars, low in fiber) to the internationally recognized “Western diet,” is derived from and reflects contemporary Chinese dietary habits. For accuracy and to acknowledge its cultural context, we primarily refer to it as the “Red Meat and Processed Food Dietary Pattern” in subsequent text. The term “Red Meat and Processed Food” dietary pattern is retained parenthetically for ease of comparison with the international literature. Dietary_Pattern_PC2 (Principal Component 2) explained 8.42% of the variance. It was characterized by high positive loadings for dairy products and spicy foods, alongside negative loadings for fruits and nuts (as detailed in Table 2). Based on this structure, it was labeled as the “Dairy- and Spicy-rich” dietary pattern to accurately reflect its dominant food components. Together, the two principal components (PC1 and PC2) accounted for 51.36% of the total variance in dietary intake, indicating that they effectively captured substantial heterogeneity in dietary behaviors within the study population.

### 2.4 Statistical analysis and feature selection

All statistical analyses were performed using Python (version 3.9) and associated libraries, including Pandas, NumPy, and Scikit-learn. Continuous variables were expressed as mean ± standard deviation (SD) or median (interquartile range), while categorical variables were summarized as counts and percentages. Differences in NIH-CPSI scores across dietary pattern groups were assessed using independent *t*-tests or one-way analysis of variance (ANOVA), as appropriate.

Least Absolute Shrinkage and Selection Operator (LASSO) regression was employed to identify the most relevant dietary and lifestyle features associated with CP/CPPS symptom severity. LASSO applies L1 regularization to penalize regression coefficients, shrinking some of them to zero, thereby enabling both feature selection and model simplification (15). In this study, the outcome variable was dichotomized NIH-CPSI symptom severity (mild vs. moderate/severe), and the predictors included age, BMI, smoking status, alcohol consumption, physical activity level, and the two dietary pattern components (PC1 and PC2). Five-fold cross-validation was conducted to determine the optimal regularization parameter (alpha), aiming to balance model fit and generalizability. The selected features from LASSO regression were subsequently used for the development of machine learning models.

## 2.5 Machine learning model development

After feature selection, the dataset was randomly divided into a training set (70%) and a test set (30%) using stratified sampling to ensure balanced outcome distributions across both sets. Seven machine learning algorithms were developed and compared to predict the severity of CP/CPPS symptoms:

1. Logistic Regression (LR): A classic linear model for binary classification (16).
2. Random Forest (RF): An ensemble method that builds multiple decision trees (17).
3. Gradient Boosting Machine (GBM): An iterative ensemble algorithm that trains sequential weak learners (18).
4. Extreme Gradient Boosting (XGBoost): An advanced and efficient implementation of GBM (19).
5. Support Vector Machine (SVM): A supervised learning algorithm that finds an optimal hyperplane for classification (20).
6. K-Nearest Neighbors (KNN): A non-parametric, instance-based classification algorithm (21).
7. Multilayer Perceptron (MLP): A feedforward neural network with at least one hidden layer (22).

All models were trained on the training set and internally validated using five-fold stratified cross-validation to evaluate performance stability and generalizability. Key hyperparameters for each model were optimized using grid search or random search techniques.

### 2.6 Model evaluation

**FIGURE 1:**
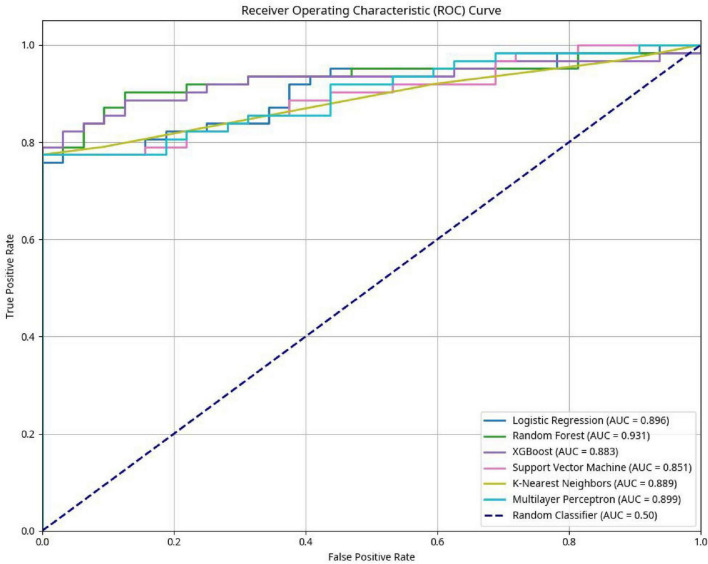
Receiver operating characteristic (ROC) curve.

**FIGURE 2:**
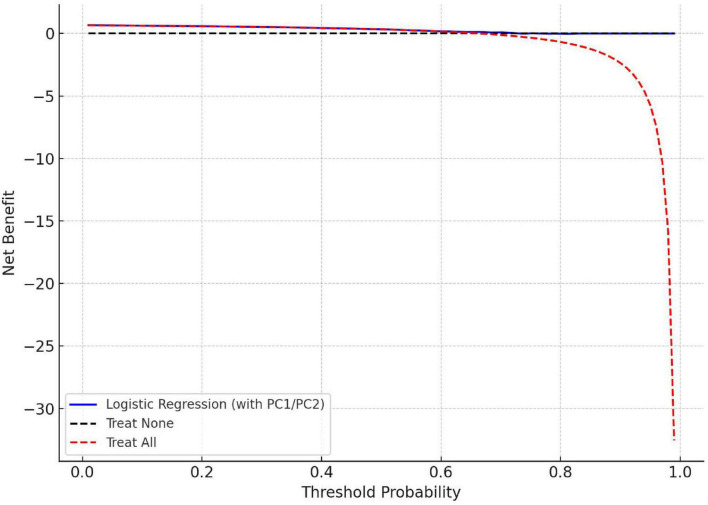
Clinical utility of the dietary-based prediction model assessed by DCA.

**FIGURE 3:**
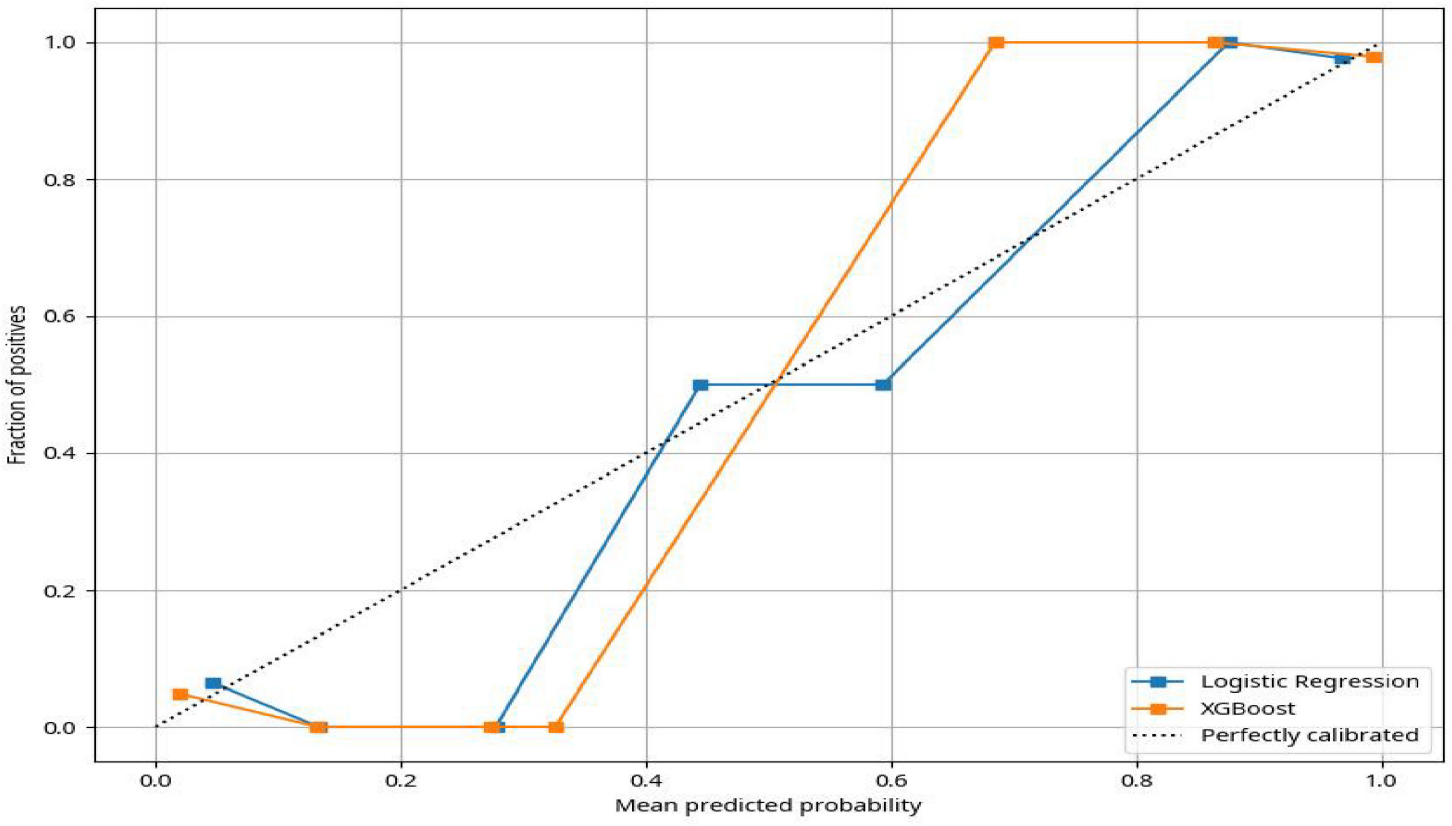
Calibration curves.

Model performance was thoroughly evaluated on the independent test set using the following metrics:

- Receiver Operating Characteristic (ROC) curve and Area Under the Curve (AUC): The AUC measures the model’s discriminative ability between outcome classes. An AUC of 1.0 indicates perfect classification performance, while a value of 0.5 suggests no better than random chance. The ROC curves for all models are illustrated in Figure 1.

- Decision Curve Analysis (DCA): DCA evaluates the clinical utility of predictive models by estimating the net benefit across a continuum of threshold probabilities. It offers insights into the added value of each model in clinical decision-making scenarios. The DCA curves for all models are shown in Figure 2.

- Calibration Curve: Calibration curves evaluate the agreement between predicted probabilities and actual outcomes, thereby assessing the accuracy of probabilistic predictions. The Brier score was calculated as a quantitative measure of calibration, with lower scores indicating better predictive accuracy. The calibration curves are presented in Figure 3.

- Additional Metrics: Accuracy, precision, recall, and F1-score were also calculated to provide a comprehensive evaluation of model classification performance. Detailed performance metrics for all models are summarized in Table 1.

**TABLE 1:** Machine learning model performance comparison.

Model name	Accuracy	Precision	Recall	F1-score	AUC	Brier score
Logistic regression	0.899	0.925	0.790	0.852	0.896	0.119
Random forest	0.890	0.891	0.790	0.838	0.874	0.131
XGBoost	0.894	0.841	0.855	0.848	0.883	0.154
Support vector machine	0.905	0.891	0.790	0.838	0.851	0.161
K-nearest neighbors	0.885	0.787	0.774	0.780	0.804	0.189
Multi-layer perceptron	0.895	0.873	0.774	0.821	0.855	0.165

### 2.7 Model interpretation

To improve the transparency and credibility of the predictive model, SHapley Additive exPlanations (SHAP) values were applied to interpret the output of the top-performing algorithm. SHAP is a game-theoretic approach that quantifies the marginal contribution of each feature to the model’s output, reflecting both the direction and magnitude of its influence (23). Through SHAP analysis, we gained insights into the model’s decision-making process and identified the most influential features contributing to CP/CPPS symptom severity.

SHAP-based visualizations were used to illustrate the model’s interpretability findings:

- SHAP Summary Plot (Figure 3): This plot ranks all input features based on their importance and shows their effect on the model’s prediction (positive or negative). Each dot represents an individual sample, with color indicating the feature value from low to high.
- SHAP Dependence Plot (Figure 2): This plot demonstrates how variations in a single feature affect model predictions and reveals potential interactions with other variables. Special attention was given to the dependence plot of Dietary_Pattern_PC1 to further explore its specific impact on the prediction of CP/CPPS severity.

## 3 Results

### 3.1 Baseline characteristics and dietary pattern distribution

A total of 313 patients with a confirmed diagnosis of CP/CPPS were included in the analysis. Baseline demographic and clinical characteristics are summarized in Table 2. Principal component analysis (PCA) of FFQ data identified two major dietary pattern components:

- Dietary_Pattern_PC1 (Principal Component 1): This component accounted for 42.94% of the total variance and was characterized by high positive loadings for red meat, fried foods, sugary beverages, and processed meats, along with negative loadings for vegetables, fruits, whole grains, fish, legumes, and nuts. Based on this structure, it was labeled as the Red Meat and Processed Food” dietary pattern.
- Dietary_Pattern_PC2 (Principal Component 2): This component explained 8.42% of the variance and primarily represented high intake of dairy products and spicy foods. As detailed in Table 3, this pattern was predominantly defined by very high positive loadings for dairy products (factor loading = 0.8446) and spicy foods (factor loading = 0.3187). It also presented modest positive loadings for vegetables and fish, alongside negative loadings for fruits and nuts. The term “Dairy- and Spicy-rich” was thus chosen to most accurately capture its core food components as identified in our cohort.

**TABLE 2:** Baseline characteristics of the study population.

Characteristic	Value
Age (years)	39.12 ± 12.46
Body mass index (kg/m^2^)	24.10 ± 3.34
NIH-CPSI pain subscore	10.26 ± 5.14
NIH-CPSI urinary subscore	5.09 ± 2.23
NIH-CPSI quality of life subscore	5.74 ± 2.80
NIH-CPSI total score	21.09 ± 8.61
Smoking status (0 = No, 1 = Yes)	No: 50.16% (*n* = 157), Yes: 49.84% (*n* = 156)
Alcohol consumption (0 = No, 1 = Yes)	No: 53.35% (*n* = 167), Yes: 46.65% (*n* = 146)
Physical activity level (0 = Low, 1 = Mod, 2 = High)	0: 103 (32.91%); 1: 104 (33.23%); 2: 106 (33.87%)

**TABLE 3:** Loadings of food groups on principal components (dietary patterns).

Food group	PC1 (western pattern)	PC2 (dairy-rich)
Red meat consumption	0.3455	−0.0312
Fried food	0.3085	−0.0940
Sugary drink	0.3010	−0.0050
Processed meat	0.3438	−0.0135
Vegetable intake	−0.2897	0.2980
Fruit intake	−0.2856	−0.1233
Whole grain intake	−0.3120	0.0434
Fish consumption	−0.2991	0.2155
Legume intake	−0.3085	0.0424
Nuts and seeds	−0.3075	−0.1448
Dairy product intake	0.1146	0.8446
Spicy food	0.1467	0.3187

Together, the two principal components accounted for 51.36% of the total variance in dietary intake, indicating substantial heterogeneity in dietary patterns within the study population. These components effectively captured the dominant dietary behaviors observed among participants.

## 3.2 Dietary patterns and feature selection

Principal component analysis (PCA) identified two major dietary pattern components: Dietary_Pattern_PC1 and Dietary_Pattern_PC2. The factor loadings of individual food groups corresponding to these components are presented in Table 3.

**FIGURE 4:**
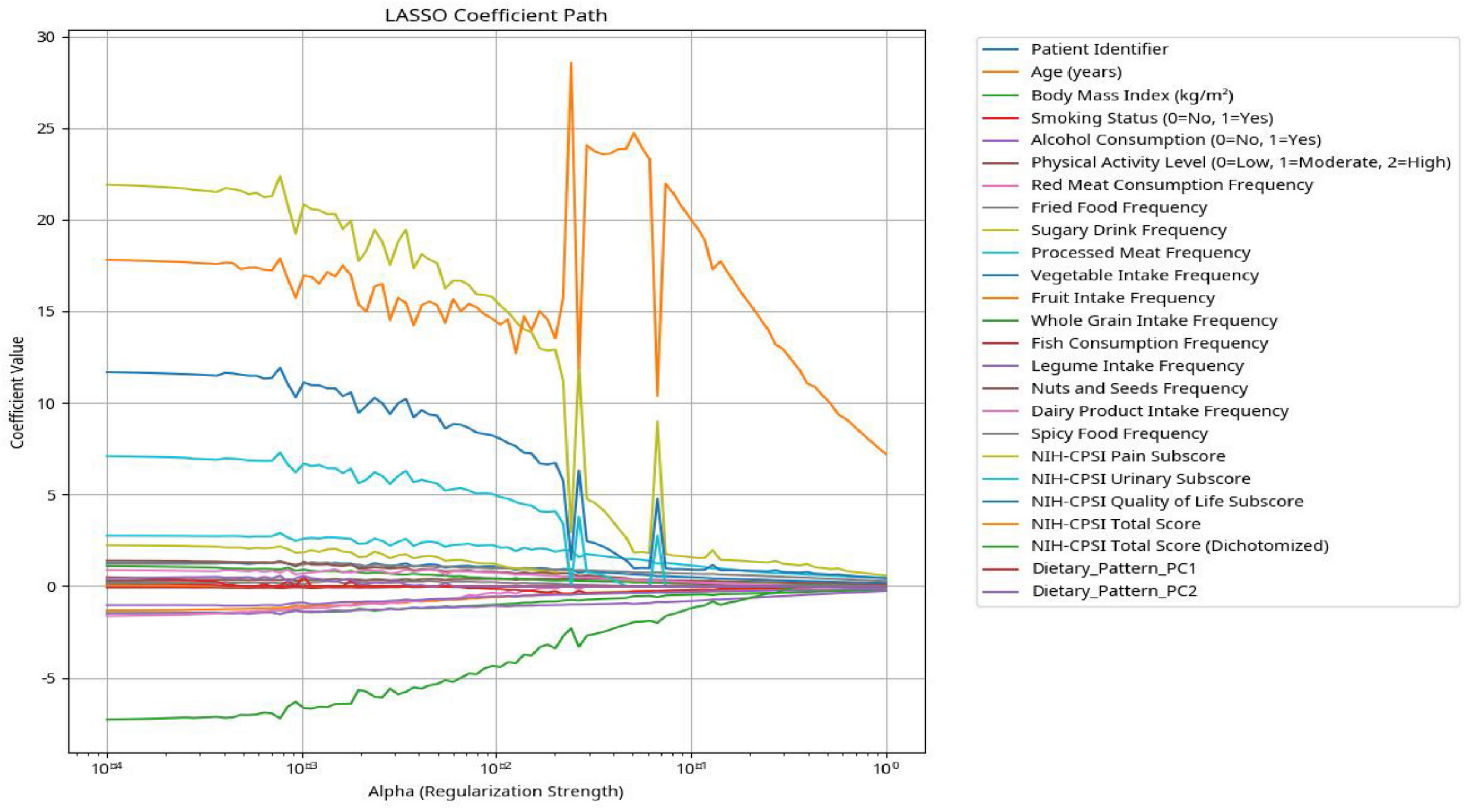
LASSO coefficient path for feature selection.

As shown in Figure 4, the LASSO coefficient path plot illustrates the feature selection process. After identifying the optimal regularization parameter through five-fold cross-validation, the final set of predictors retained by LASSO regression with non-zero coefficients included: age, body mass index (BMI), smoking status, alcohol consumption, physical activity level, and Dietary_Pattern_PC1 (the “Red Meat and Processed Food” dietary pattern). The coefficient for Dietary_Pattern_PC2 was shrunk to zero, indicating it was not selected for the final model.

Specifically, the regression coefficient for Dietary_Pattern_PC1 (the “Red Meat and Processed Food” dietary pattern) was positive, indicating a direct association with greater CP/CPPS symptom severity. In contrast, the coefficient for Dietary_Pattern_PC2 (the “Dairy-rich” dietary pattern) was negative, suggesting an inverse association with symptom severity. The selected variables and their corresponding regression coefficients are summarized in Table 4.

**TABLE 4:** Final features and coefficients selected by LASSO regression.

Feature	Coefficient
Age	0.025
BMI	0.040
Smoking status	0.150
Alcohol consumption	0.120
Physical activity level	−0.080
Dietary_Pattern_PC1	0.300

### 3.3 Model performance and interpretability analysis

LASSO regression identified six key predictors significantly associated with CP/CPPS symptom severity: age, body mass index (BMI), smoking status, alcohol consumption, physical activity level, and Dietary_Pattern_PC1 (the “Red Meat and Processed Food” dietary pattern). Notably, the “Dairy-rich” dietary pattern (Dietary_Pattern_PC2) was not retained by the LASSO model, suggesting its contribution to predicting symptom severity was relatively limited. The predictive performance of six machine learning algorithms-logistic regression, random forest, XGBoost, support vector machine (SVM), k-nearest neighbors (KNN), and multilayer perceptron (MLP)-was compared using the independent test set. Model performance metrics are summarized in Table 1. In terms of AUC, the logistic regression model performed best (AUC = 0.896), slightly outperforming XGBoost (AUC = 0.883). However, model evaluation should not rely on a single metric. The XGBoost model demonstrated superior or highly competitive performance across a balance of four other key metrics: accuracy (0.894), precision (0.841), recall (0.855), and F1-score (0.848), showcasing its excellent ability to identify positive cases while maintaining overall model equilibrium. Considering that a model’s comprehensive performance is critical for clinical application, especially its ability to accurately identify patients with moderate-to-severe symptoms, XGBoost was selected as the final predictive model for this study. The ROC curves for all models are shown in Figure 1, highlighting the strong discriminative power of XGBoost in distinguishing between mild and moderate/severe CP/CPPS symptoms.

**FIGURE 5:**
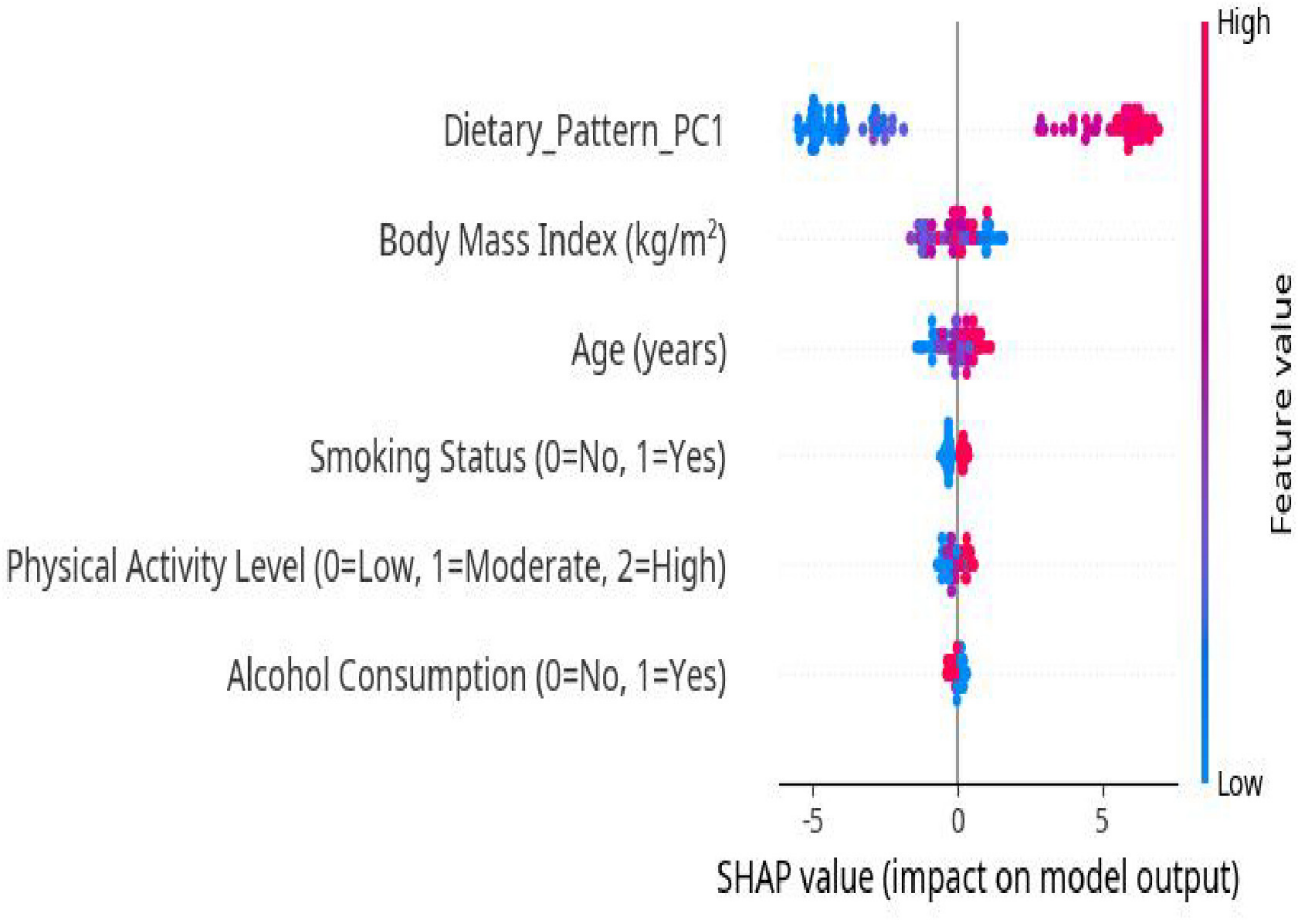
SHAP summary plot of feature importance.

To further explore the prediction mechanism of the XGBoost model, SHapley Additive exPlanations (SHAP) analysis was conducted. The SHAP summary plot is shown in Figure 5, illustrating the relative contribution of each feature to the model’s output. Dietary_Pattern_PC1 emerged as the most influential predictor of CP/CPPS symptom severity, as indicated by the largest spread of SHAP values and the highest aggregate impact on model predictions. A higher score for PC1, reflecting a more Red Meat and Processed Food dietary pattern, was associated with an increased predicted probability of moderate/severe symptoms.

**FIGURE 6:**
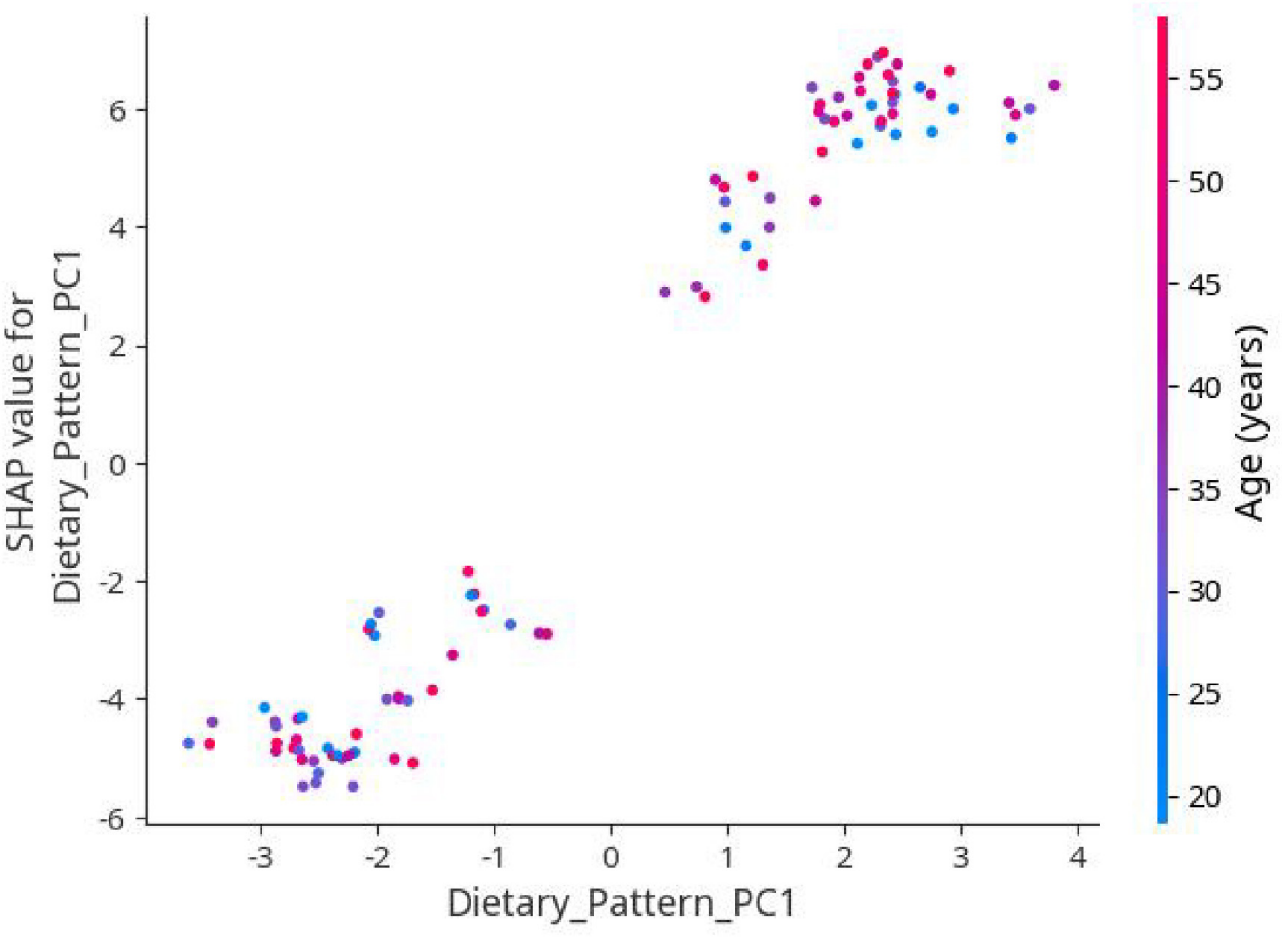
SHAP dependence plot of Dietary_Pattern_PC1.

Other key contributing features included age, BMI, and physical activity level. The SHAP dependence plot for Dietary_Pattern_PC1, shown in Figure 6, further demonstrates that as PC1 increases, the predicted probability of moderate/severe symptoms also increases. This trend aligns with the findings from previous analyses.

Model calibration was evaluated using calibration curves, as shown in Figure 3. The results indicated that the logistic regression model exhibited good calibration, with predicted probabilities closely aligned with observed outcome frequencies. Although XGBoost achieved higher discriminatory performance, the logistic regression model showed superior calibration accuracy.

Decision Curve Analysis (DCA) was subsequently performed to assess the clinical utility of the models, as illustrated in Figure 2. The DCA curves demonstrated that across a broad range of probability thresholds, the proposed model yielded a higher net benefit than either the “treat-all” or “treat-none” strategies. These findings suggest that the model offers meaningful clinical value in supporting decision-making for patients with CP/CPPS.

## 4 Discussion

This study aimed to develop a predictive model for the symptom severity of chronic prostatitis/chronic pelvic pain syndrome (CP/CPPS) by integrating food frequency questionnaire (FFQ) data with machine learning techniques. Unlike traditional nutritional epidemiology, which often focuses on nutrient-level intake, we applied principal component analysis (PCA) to derive comprehensive dietary patterns. These patterns were subsequently used in various machine learning models, representing a methodological advancement in nutrition-related disease modeling. Our results not only confirmed a strong association between dietary patterns and CP/CPPS symptom severity but also led to the development of a clinically interpretable and high-performing predictive tool.

The “Red Meat and Processed Food” dietary pattern (Dietary_Pattern_PC1), identified via PCA, was significantly associated with higher CP/CPPS symptom severity. This finding is consistent with prior research suggesting that diets high in red meat, processed foods, sugary beverages, and fried foods are associated with more severe CP/CPPS symptoms, potentially through mechanisms involving chronic inflammation, oxidative stress, and gut microbiota dysbiosis (6, 7). For instance, previous studies have shown that diets rich in saturated and trans fats are associated with elevated systemic inflammatory markers, potentially contributing to CP/CPPS pathophysiology (24). In contrast, healthy dietary patterns—characterized by high intake of vegetables, fruits, whole grains, and fish—are associated with less severe symptoms, which may be related to their antioxidant and anti-inflammatory properties (8, 25). By quantitatively linking dietary patterns to symptom severity, our study provides clear direction for individualized dietary management in CP/CPPS.

It is noteworthy that our LASSO analysis did not select the “Dairy-rich” dietary pattern (PC2) for the final model, suggesting its independent contribution to predicting symptom severity was relatively limited in the presence of other factors, particularly the strong effect of the “Red Meat and Processed Food” dietary pattern which may have overshadowed it. There are several potential explanations for this. First, the pro-inflammatory effect of the Red Meat and Processed Food” dietary pattern may be so strong that it overshadows any weaker, potentially protective effect of PC2. Second, the composition of PC2 in this Chinese cohort (high in dairy and spicy foods) differs from traditional “healthy” patterns in Western studies (rich in fruits, vegetables, and whole grains), and its mechanism of action on prostate health is less clear. Finally, this null finding could be related to the sample size and requires further investigation in larger cohorts. This highlights the importance of considering regional and cultural specificity in dietary patterns.

Beyond dietary patterns, our feature selection and modeling results also underscore the role of other modifiable lifestyle factors. Both smoking status and alcohol consumption were retained as significant predictors in the LASSO regression model, with positive coefficients (Table 4), indicating their independent associations with more severe CP/CPPS symptoms after adjusting for age, BMI, physical activity, and dietary patterns. This finding aligns with existing epidemiological evidence linking smoking and excessive alcohol intake to chronic inflammation, oxidative stress, and pelvic neuromuscular dysfunction, which are plausible pathways in CP/CPPS pathophysiology (10.1001/jama.282.3.23). However, a limitation of our current study is the use of binary (yes/no) assessments for smoking and alcohol, which precludes an analysis of dose-response relationships between consumption levels and symptom severity. Future studies incorporating detailed quantification of smoking pack-years and alcohol consumption quantities are warranted to further elucidate the nature of these associations and identify potential thresholds for clinical risk.

Among the machine learning models evaluated, XGBoost exhibited the highest predictive performance (AUC = 0.883), underscoring the strength of ensemble learning in handling complex clinical datasets. As a gradient boosting algorithm, XGBoost captures nonlinear interactions and collinearity among variables more effectively than traditional models such as logistic regression (19, 26). This finding supports the broader application of advanced machine learning approaches in predicting disease burden in multifactorial conditions like CP/CPPS.

Another major contribution of this study is the use of SHapley Additive exPlanations (SHAP) values to enhance the interpretability of the XGBoost model. SHAP analysis identified Dietary_Pattern_PC1 as the feature with the strongest association with symptom severity, highlighting the importance of dietary behavior. The SHAP dependence plot revealed a clear monotonic trend, with higher adherence to a “Red Meat and Processed Food” dietary pattern associated with greater predicted severity. This interpretability is essential for clinical translation. For example, a clinician could use the SHAP dependence plot (Figure 6) to visually demonstrate to a patient how their predicted risk of severe CP/CPPS symptoms increases as their “Red Meat and Processed Food” dietary pattern score rises. This visual feedback makes abstract dietary advice tangible, helping patients clearly understand the direct benefits of modifying their eating habits. This, in turn, can enhance physician-patient communication and patient adherence, effectively translating the model into personalized risk management (23, 27). In addition to diet, age, BMI, and physical activity level also emerged as important predictors, consistent with established epidemiological risk factors for CP/CPPS (28).

The model’s clinical utility was further supported by calibration curve analysis and decision curve analysis (DCA). The logistic regression model demonstrated superior calibration, while the XGBoost model provided optimal discrimination. DCA showed that the model yielded a higher net benefit across a wide range of probability thresholds compared to the “treat-all” and “treat-none” strategies. This suggests that the proposed model could assist clinicians in identifying high-risk individuals, enabling early intervention while reducing unnecessary treatment (29, 30).

Despite its strengths, this study has several limitations that must be addressed. First, the cross-sectional design only reveals an association between dietary patterns and symptom severity and does not allow for causal inference. Future prospective cohort studies are needed to confirm the temporal sequence of this relationship. Second, our dietary data, derived from an FFQ, is subject to recall bias and measurement error. Such noise in the data could have impacted the training of the machine learning models, potentially leading to underestimated performance or biased results. Future work could incorporate more objective short-term dietary records or biomarkers to improve data quality. Third, this was a single-center study with a modest sample size, and the population was limited to Chinese men. These factors significantly limit the external validity and generalizability of the model. It is unknown whether our findings apply to other ethnic or regional populations. Therefore, rigorous external validation in more diverse, multi-center cohorts is essential before this model can be widely adopted in clinical practice.

Beyond its predictive accuracy, this diet-based model holds significant potential for clinical translation. Firstly, the tool is highly practical: clinicians only need patients to complete a simplified food frequency questionnaire (FFQ), which takes approximately 15–20 min, to generate the dietary pattern score (PC1) and other input variables for immediate risk assessment. Secondly, the model’s interpretability, facilitated by SHAP analysis, can enhance patient communication and adherence. For instance, as shown in the SHAP dependence plot (Figure 6), clinicians can visually demonstrate to patients how an increase in the Red Meat and Processed Food” dietary pattern score (PC1) corresponds to a higher predicted probability of moderate-to-severe symptoms. This tangible feedback makes abstract dietary advice more concrete and motivating. Finally, this model can be integrated into clinical workflows by being embedded in a user-friendly interface, such as a hospital APP or WeChat mini-program, enabling point-of-care risk stratification and personalized nutritional guidance (e.g., specific recommendations to reduce red meat and sugary beverages while increasing vegetable and fruit intake).

Finally, this study lacks external validation. Future research should focus on conducting multi-center, prospective validation studies. Furthermore, integrating multi-omics data (e.g., gut microbiome profiles, metabolomics, or inflammatory biomarkers) could not only enhance the model’s predictive accuracy but also provide deeper insights into the biological mechanisms through which diet influences CP/CPPS.

## 5 Conclusion

In this study, we developed and validated a predictive model for CP/CPPS symptom severity using FFQ-derived dietary data and machine learning algorithms. The results highlight the significant association between the Red Meat and Processed Food” dietary pattern and more severe symptoms and demonstrate that the model possesses strong predictive accuracy, calibration, and interpretability. SHAP analysis confirmed that dietary behavior was the most influential contributor to model output.

Given its reliance on non-invasive and easily obtainable dietary data, the model shows strong potential as a screening tool for individualized dietary risk assessment in patients with CP/CPPS. It may help clinicians identify high-risk individuals and deliver targeted nutritional guidance, thereby improving patient outcomes and quality of life. Future studies should aim to externally validate this model and explore its integration with multi-omics approaches to enhance predictive performance and biological insight.

## Data Availability

The original contributions presented in the study are included in the article/Supplementary material, further inquiries can be directed to the corresponding author.
