# Supplementary material for: Dietary patterns and chronic prostatitis: a symptom severity prediction model based on nutritional clustering and machine learning
Source: Front Nutr. 2026 Jan 19;12:1660430. doi: 10.3389/fnut.2025.1660430 (PMC12863200; doi:10.3389/fnut.2025.1660430)
Supplement: Supplementary file 1 [file Table_1.docx]

| Variable | Encoding |
| --- | --- |
| Smoking Status | 0=No, 1=Yes |
| Alcohol Consumption | 0=No, 1=Yes |
| Physical Activity Level | 0=Low, 1=Moderate, 2=High |
| Red Meat Consumption Frequency | 0=Never, 1=Occasionally, 2=Weekly, 3=Daily |
| Fried Food Frequency | 0=Never, 1=Occasionally, 2=Weekly, 3=Daily |
| Sugary Drink Frequency | 0=Never, 1=Occasionally, 2=Weekly, 3=Daily |
| Processed Meat Frequency | 0=Never, 1=Occasionally, 2=Weekly, 3=Daily |
| Vegetable Intake Frequency | 0=Never, 1=Occasionally, 2=Weekly, 3=Daily |
| Fruit Intake Frequency | 0=Never, 1=Occasionally, 2=Weekly, 3=Daily |
| Whole Grain Intake Frequency | 0=Never, 1=Occasionally, 2=Weekly, 3=Daily |
| Fish Consumption Frequency | 0=Never, 1=Occasionally, 2=Weekly, 3=Daily |
| Legume Intake Frequency | 0=Never, 1=Occasionally, 2=Weekly, 3=Daily |
| Nuts and Seeds Frequency | 0=Never, 1=Occasionally, 2=Weekly, 3=Daily |
| Dairy Product Intake Frequency | 0=Never, 1=Occasionally, 2=Weekly, 3=Daily |
| Spicy Food Frequency | 0=Never, 1=Occasionally, 2=Weekly, 3=Daily |
| Symptom Severity | 0=Mild, 1=Moderate/Severe |

Supplementary Table S1
